# Detection of S-HBsAg Mutations in Patients with Hematologic Malignancies

**DOI:** 10.3390/diagnostics11060969

**Published:** 2021-05-27

**Authors:** Maria V. Konopleva, Maxim S. Belenikin, Andrei V. Shanko, Alexey I. Bazhenov, Sergei A. Kiryanov, Tatyana A. Tupoleva, Maria V. Sokolova, Alexander V. Pronin, Tatyana A. Semenenko, Anatoly P. Suslov

**Affiliations:** 1 Federal State Budget Institution “National Research Centre for Epidemiology and Microbiology Named After Honorary Academician N.F. Gamaleya” of the Ministry of Health of the Russian Federation, 123098 Moscow, Russia; maria-konopleva@rambler.ru (M.V.K.); shankoandrei@gmail.com (A.V.S.); sergeisashilov@hotmail.com (S.A.K.); sokolova_mariya_gamaleya@mail.ru (M.V.S.); proninalexander@yandex.ru (A.V.P.); semenenko@gamaleya.org (T.A.S.); 2 Laboratory of Molecular Medical Diagnostics, Moscow Institute of Physics and Technology, State University, 141701 Dolgoprudny, Russia; molecular.modeler@gmail.com; 3 State Budget Institution “Research Institute of Emergency Medicine Named After N.V. Sklifosovsky” of the Moscow Department of Healthcare, 129010 Moscow, Russia; albazhenov@yandex.ru; 4 National Research Center for Hematology, 125167 Moscow, Russia; tupoleva.t@blood.ru

**Keywords:** mutation, S-gene, HBV, HBsAg, escape, oncohematology, lymphoma, leukemia

## Abstract

Multiple studies of hepatitis B virus (HBV) genetic variability and its relationship with the disease pathogenesis are currently ongoing, stemming from growing evidence of the clinical significance of HBV mutations. It is becoming increasingly evident that patients with hematologic malignancies may be particularly prone to a higher frequency of such mutations. The present report is the first extensive study of the prevalence of escape mutations in S-HBsAg, performed using isolates from 59 patients from hospital hematology departments with diagnoses of leukemia (n = 32), lymphoma (n = 20), multiple myeloma (n = 3), and non-tumor blood diseases (n = 4). The isolates were serologically examined for the presence of HBV markers and sequenced using either next-generation sequencing (NGS) or Sanger sequencing. Occult hepatitis B was found in 5.1% of cases. Genetic analysis of the region corresponding to S-HBsAg demonstrated an exceptionally high mutation frequency in patients with leukemias (93.4%) and lymphomas (85.0%), along with the prominent mutation heterogeneity. Additionally, more than 15 mutations in one sample were found in patients with leukemias (6.3% of cases) and lymphomas (5.0% of cases). Most of the mutations were clinically significant. The study analyzes the mutation profile of HBV in different oncohematological diseases and the frequency of individual mutations. The data strongly suggest that the NGS method, capable of detecting minor populations of HBV mutations, provides a diagnostic advantage, lays the foundation for the development of screening methods, and allows for the study of the virological and pathogenetic aspects of hepatitis B.

## 1. Introduction

The emergence of immunological escape mutations is one of the urgent problems associated with hepatitis B. These mutations generally occur in the main hydrophilic loop of the a-determinant region of the hepatitis B virus surface antigen (HBsAg). Hepatitis B virus (HBV) is a DNA virus; however, it has a complex replication cycle involving a reverse transcriptase [1], which determines the HBV high variability comparable to that of retroviruses [2,3]. Measuring the frequency of nucleotide sequence substitutions in the HBV genome is hampered by the existence of overlapping reading frames and the absence of similar sites in most coding regions. However, it is believed that the HBV mutation rate is more than ten times higher than in most other DNA viruses. Furthermore, the number and prevalence of HBV mutants in patients’ sera varies as a function of time [4]. The frequency of nucleotide substitutions in the HBV genome also varies depending on the stage of the disease. In chronic hepatitis B, the natural evolutionary frequency of mutations is approximately 1.4 × 10^−5^ substitutions per site per year [5], whereas in patients that receive liver transplants, this value increases by two orders of magnitude [6]. The emergence of immunological escape mutations can be triggered by vaccination, immunoglobulin therapy or the functional state of the patient’s immune system, for example, due to immunosuppression [7]. Under the pressure of various selection factors, HBV evolves as a pool of quasispecies, mutants, and genotypes, which provides the virus with a significant survival advantage [3]. 

The strategy of large-scale vaccination against hepatitis B, introduced in most countries, has significantly reduced the global prevalence of HBV; however, an accumulation of HBV escape mutants, especially among vaccinated children, is observed worldwide. G145R is the most significant mutation [8,9,10,11,12]. It has been shown that the G145R mutant can be present in patients in the form of a minor population [13] and, at the same time, be transmitted vertically [14]. Hence, the true prevalence of such mutants is underestimated. On the other hand, our studies have previously shown that the widely used hepatitis B vaccines do not elicit a cross-protective immunity against the G145R mutant [15]. Thus, the potential for the spread of mutant viral strains is accumulating. Existing mathematical models predict the likelihood for the G145R mutant to replace the dominant variant of HBV [16,17]. 

Previously, multiple publications have reported on the failure of commercial serological test systems to detect HBsAg mutants of HBV. These included reports on HBsAg-negative carriers in which HBV was detected only by the presence of viral DNA, or carriers with unusual profiles of serological markers of HBV infection [7,18]. The latter include HBV carriers who only have a hepatitis B core antibody (anti-HBc) in their blood; patients with discordant test results in various immunodiagnostic systems; individuals with negative HBsAg test results and positive hepatitis B e-antigen (HBeAg) test results; patients negatively tested for HBsAg but positively tested for anti-HBs and anti-HBc (serological confirmation of previous HBV infection); finally, infected people who simultaneously have circulating HBsAg and anti-HBsAg serum antibodies, typically in concentrations not exceeding 100 mU/mL. Such combinations of diagnostic markers are often associated with occult HBV infection (OBI) [3,19,20,21,22]. 

OBI is considered a high potential risk factor for the development of hepatitis, liver cirrhosis, post-transfusion hepatitis (PTH), and hepatocellular carcinoma (HCC) [22]. Some authors suggested that the development of OBI may be associated with escape mutations in the S-gene of HBV [22,23], although this is not proven [22]. Nevertheless, occult hepatitis B is a well-known complication in patients with chronic HBV infection receiving cytotoxic or immunosuppressive therapy, including hematological cancer patients [24]. During immunosuppression, viral reactivation may occur in patients previously demonstrating markers of occult hepatitis B, leading to severe acute hepatitis, fulminant liver failure, and death [25]. Reactivation of HBV infection has been reported in diseases such as leukemias and lymphomas, as well as following bone marrow and hematopoietic stem cell transplantation [26,27]. The substitutions in amino acid residues (aa) 145, 147, and 123 of HBsAg are most often considered immunological escape mutations associated with OBI [22].

Taken together, the available literature data suggest the importance of establishing risk groups in which the frequency of detected HBV escape mutants is higher than the population average. Hematological cancer patients who are immunosuppressed are such a risk group. Previously, we have frequently detected serological markers of HBV infection among patients in hospital hematology departments who were not vaccinated against hepatitis B, compared with blood donors, and those patients often had occult hepatitis B [21,28]. When sequencing the S-gene by the Sanger method, substitutions were detected in 75.0% of patients in hematology departments [21].

The problem of reduced sensitivity of serological diagnostics of HBsAg escape mutants has been largely resolved. For example, a comparative study of three point-of-care (POC) tests and the ARCHITECT test system for detecting HBsAg with escape mutations demonstrated that the sensitivity of the POC tests varied from 98 to 100%, with only a single false-negative D144A mutation [29]. Despite the high sensitivity and specificity of modern enzyme-linked immune assays for detecting HBsAg, they are unable to differentiate between immunological escape mutants, especially those that make up a small population of quasispecies.

The aim of the present study was to assess the prevalence of HBV escape mutants, including minor variants, in a group of patients with chronic hepatitis B and malignant blood diseases, by capillary sequencing or next-generation sequencing (NGS).

## 2. Materials and Methods

### 2.1. Patient Groups and Serum Samples

The study included 59 patients with chronic hepatitis B who were treated in specialized oncohematological departments of Moscow hospitals. The gender proportion was 66.0% (men) and 34.0% (women). The average age of the subjects was 50.3 ± 18.3 years, 52.6 ± 16.8 for men and 48.1 ± 18.9 for women. Thus, men and women did not differ statistically significantly by age, *p* = 0.488 based on the Student t-test. Informed voluntary consent was obtained from all study subjects who participated in this study according to the ethical principles laid in the World Medical Association’s Declaration of Helsinki.

The experimental group “Leukemias” (n = 32) contained samples from patients with the following diagnoses: acute leukemia (AL, n = 4), acute lymphoblastic leukemia (ALL, n = 2), acute myeloid leukemia (AML, n = 12), acute myelomonoblastic leukemia (AMML, n = 2), chronic lymphoblastic leukemia (CLL, n = 11), and chronic myeloid leukemia (CML, n = 1).

The experimental group “Lymphomas” (n = 20) included samples from patients with the following diagnoses: lymphogranulomatosis or Hodgkin’s lymphoma (HL, n = 12), lymphosarcoma or non-Hodgkin’s lymphoma (NHL, n = 1), lymphoma (n = 5), B-cell lymphoma (n = 1), and T-cell lymphoma (n = 1).

The experimental group “Multiple myeloma” included samples from 3 patients with this diagnosis.

The experimental group “Non-neoplastic” included samples from 4 patients with mixed diagnoses not related to malignant tumors: myelodysplastic syndrome (MDS, n = 1), aplastic anemia (AA, n = 2), and idiopathic thrombocytopenic purpura (ITP, n = 1).

### 2.2. Commercial Diagnostic Kits

Serum samples were examined for the presence of serological markers of HBV (HBsAg, anti-HBs, HBeAg, anti-HBe IgG, anti-HBc IgM+IgG). In addition, the HBV viral load was determined in the samples.

Assessment of blood sera for the presence of HBsAg was performed using the enzyme-linked immune test system "Hepastrip B" (NEARMEDIC PLUS LLC, Moscow, Russia). Testing for other markers of HBV infection was performed by enzyme-linked immunosorbent assay (ELISA) using test systems manufactured by JSC “Vector-Best”, Novosibirsk, Russia: “VectoHBe-IgG” (cat. No. D-0578), “Vecto-HBe antigen” (cat. No. D-0576), "Vecto-HBc antibodies" (cat. No. D-0566), and “Vecto-HBsAg antibodies” (cat. No. D-0562). The ELISA was performed according to the manufacturer’s instructions. 

Isolation and quantitative determination of HBV DNA was performed by real-time PCR using a set of reagents "RealBest DNA HBV" (quantitative version) (cat. No. D-0599, JSC “Vector-Best”, Novosibirsk, Russia), according to the manufacturer’s instructions.

### 2.3. Partial Sanger Sequencing of the S-gene

Partial Sanger sequencing of the S-gene was carried out for 17 isolates.

Viral DNA was isolated using SDS/EDTA with protein kinase K and alkaline phenol extraction [30]. HBV DNA was amplified by nested PCR in two steps. In the first stage, a forward primer was HBV-S1 (5′-CTCGTGTTACAGGCGGGGTTTTTC-3′) and a reverse primer was HBV-S2 (5′-CATCATCCATATAGCTGAAAGCCAAACA-3′. In the second stage, a forward primer was HBV-S3 (5′-TTGTTGACAAGAATCCTCACAATACC-3′) and a reverse primer was HBV-S4 (5′-GCCCTACGAACCACTGAACAAATGG-3′). The conditions for both rounds of PCR were as follows: one cycle at 95 °C for 3 min, followed by another 30 cycles (95 °C 45 s, 56 °C 1 min, 72 °C 1 min), and the final stage at 72 °C for 1 min.

Determination of the nucleotide sequences of PCR products was carried out using the ABI PRISM Big Dye TM v.1.1 kit (Applied Biosystems, Foster City, CA, USA) and an ABI-3100 PRISM Genetic Analyzer capillary automatic sequencer (Applied Biosystems, Foster City, CA, USA), according to the manufacturer’s instructions. Alignment of the S-gene nucleotide sequences and comparative analysis of the primary nucleotide sequence were performed using the Vector NTI Advance^TM^ 10.3.0 program (Thermo Fisher Scientific Inc. (Invitrogen), Waltham, MA, USA). Sequences of HBV genomes (genotypes A–H) obtained from the GenBank were used as reference data. This study analyzes the portion of the S gene corresponding to S-HBsAg.

### 2.4. Deep Sequencing of the Full-Length Genome in HBV Isolates

The study of the full-length genome was performed for 42 HBV isolates using NGS.

Amplification of DNA samples was carried out using primers located in conserved regions of the genome, taking into account the overlap of the amplified loci. Each amplification reaction was carried out separately. 

The following primers were used in the reaction [31,32]: pair 1: 1-TCACCATATTCTTGGGAACAAGA, 2-CGAACCACTGAACAAATGGC; pair 2: 1-GCCATTTGTTCAGTGGTTCG, 2-TGGGCGTTCACGGTGGT; pair 3: 1-ACCACCGTGAACGCCCA, 2-TCTTGTTCCCAAGAATATGGTGA. The resulting three PCR products were 1103 base pairs (bp), 946 bp, and 1226 bp long, respectively.

Following amplification, the PCR products were subjected to the agarose gel electrophoresis. DNA concentration in the resulting amplicons was measured using a Qubit 2.0 fluorometer (Thermo Fisher Scientific Inc. (Invitrogen), Waltham, MA, USA). The amplicons 1, 2, and 3 were then mixed equimolarly in one sample, and the amount of DNA in the pooled amplified samples was measured using a Qubit 2.0 fluorometer. The DNA concentration in the samples was normalized to 15 ng/μL. To prepare indexed libraries, 100 ng of each sample was taken into the reaction. Libraries were prepared according to the manufacturer’s standard protocol.

NGS sequencing was carried out using the Ion PMG platform (Thermo Fisher Scientific Inc. (Life Technologies), Waltham, MA, USA), using 316-type chips according to a standard protocol. Each chip included 16 pre-prepared indexed genomic libraries. While the theoretical design capacity of a 316-type chip is 300 Mbp to 1 Gbp, i.e., from 300 million to 1 billion nucleotides, we assumed a practical capacity of 500 M. The length of the HBV genome is approximately 3200 bp [31]. When simultaneously sequencing 16 indexed genomic libraries, the estimated sequencing depth was approximately 9700 reads per sample. The sequencing results showed that the average reading depth of different samples was ~1000–10,000×.

Alignment of the S-gene nucleotide sequences and comparative analysis of the primary nucleotide sequence were performed using the Vector NTI Advance^TM^ 10.3.0program (Thermo Fisher Scientific Inc. (Invitrogen), Waltham, MA, USA). Sequences of HBV genomes (genotypes A–H) obtained from GenBank were used as reference data. Determination of genotypes and subtypes of HBV, as well as the presence of mutations, was carried out as in previous reports [7,33,34]. For sequence alignment, the following references from GenBank were also used: JX096956 (Latvia, subgenotype D2) and X98077, subtype adw [35]. This study analyzes the portion of the S gene corresponding to S-HBsAg.

To identify a mutation, the genotype and subtype of the virus were first determined, followed by assessment of an amino acid substitution within the established subtype. If an amino acid variant in a subtyping determinant has been reported in the literature as common for the established subtype, it was not considered a mutation. In contrast, the heterogeneity, i.e., the simultaneous presence of both typical and atypical amino acid substitutions for a given genotype/subtype, was considered a mutation. The very fact of mutation detection was taken into account during data analysis.

### 2.5. Statistical Analysis

Experimental data processing and graph preparation were performed using the Excel 2007 (Microsoft Corporation, Redmond, Washington, United States) and GraphPad Prism 6.01 (GraphPad Software, San Diego, CA, USA) software.

## 3. Results

### 3.1. General Serological Characteristics of the Samples

Of the 59 tested sera, three samples (5.08%) were seronegative for HBsAg, indicative of occult hepatitis B (Table 1). The percentage increases to 5.45% if calculated for the hemoblastosis group. One of these samples (sample 55, lymphoma) was completely negative for all serological markers except HBV DNA, while the DNA level was very low (<10^2^ copies/mL). Another sample (sample 53, AML), also with low HBV DNA (<10^2^ copies/mL), contained anti-HBs, anti-HBe, and anti-HBc, but was negative for HBeAg. The third sample (sample 22, CLL), negative not only for HBsAg but also for anti-HBs, had a high titer of the viral DNA (about 10^8^ copies/mL), HBeAg, anti-HBeAg, and anti-HBc.

Fifty HBsAg-positive samples were tested for anti-HBs, and only two (4%) were found positive. Both of these samples contained high titers of HBV DNA as well as anti-HBc and anti-HBe, but were negative for HBeAg (Table 1).

Testing for HBeAg was performed for 52 HBsAg-positive samples, and 24 samples (46.2%) were found positive. Among HBsAg/HBeAg-positive samples, only 13 (54.2%) had anti-HBe. At the same time, the proportion of anti-HBe-positive samples among all HBsAg-positive sera tested for this trait was 72.9%. The proportion of anti-HBc-positive among HBsAg-positive samples was higher (80.8%; Table 1).

### 3.2. Genotypes, Subtypes, and Mutations in Subtyping Determinants

The present study has found that genotype D was the dominant HBV genotype (54/59 samples, 91.53%), with the subgenotype D2 with subtype ayw3 in 12 out of 59 cases. Three out of 59 samples (5.08%) belonged to genotype A and had subtypes adw1 and ayw3. In addition, one recombinant with genotype D/E was identified, and one sample was not genotyped due to incomplete sequencing (Table 1). 

Overall, mutations in S-HBsAg were found in 91.5% of all tested samples, in 93.4% of leukemia cases, and in 85.0% of lymphoma cases (Table 1). A characteristic feature for the leukemia and lymphoma groups was an extremely high mutation rate. In particular, more than 15 mutations in the same sample were detected in 6.3% of leukemia cases and 5.0% of lymphoma cases (Table 2).

Subtyping HBV aa are located in the a-determinant of HBsAg (aa 124–147), which includes the dominant HBsAg epitopes targeted by neutralizing B-cell responses. Moreover, the main hydrophilic region (MHR), exposed on the outer surface of the virion, is located between 99–169 aa of S-HBsAg [36]. Therefore, changes in subtyping determinants inevitably affect the conformation of the molecule and the B-cell epitopes of the virus. In samples from hematological cancer patients, we found high HBV heterogeneity often associated with changes in subtyping aa, which resulted in mixed subtypes. In some cases, due to changes in subtyping aa positions, the subtype of the virus could not be definitively established (Table 1, Table 3 and Table 4). Such substitutions have been described previously and considered as escape mutations of clinical significance, occasionally associated with the HBV genotype.

Among the substitutions found in the subtyping determinants are the homogeneous substitution R122T and the heterogeneous substitution R122K (quantity of R122K is about 66%). The R122T substitution does not allow for establishing the base HBV subtype ad or ay, while the R122K substitution leads to the persistence of both base subtypes in the same patient. A similar mutation, K122I, produces more glycosylated HBsAg compared to wild type [37], which alters HBsAg antigenicity and immunogenicity and affects its clearance in vivo [38]. The K122I substitution in HBsAg was common in HBsAg-negative patients with chronic HBV infection; mutations K122M and K122N were also observed [38]. Interestingly, a patient with leukemia (CML; sample 45) was found to contain not only the R122T substitution but also substitutions in the subtyping determinants that determine the w variant (the T\L\P127I substitution and the Y134N substitution), as well as many other mutations. The genotype could not be reliably established for sample 45 (Table 1 and Table 4). 

In the S-HBsAg subtyping determinant aa 127, there was an atypical T127S substitution (population quantity is about 58%), accompanied by a Y134N substitution in another subtyping determinant in a minor population (quantity is about 21%) (CLL; sample 15). Variations at S-HBsAg aa 134 included substitutions Y134F, Y134N, and Y134P and were found in 12 out of 59 samples (20.34%), often in the form of heterogeneous populations, and were not associated with either the HBV genotype or its subtype (Table 1, Table 3 and Table 4). Such variations were more common in patients with leukemias (eight out of 12 samples, 66.67%) than in patients with non-Hodgkin’s lymphoma (three out of 12 samples, 25%). Such a substitution was also found in a single case of idiopathic thrombocytopenic purpura (ITP, 8.33%). Substitutions at S-HBsAg aa 127 and aa 134 are considered immunological escape mutations [36].

In a sample from a leukemic patient with CLL with a mixed genotype D/E, a K160N mutation was identified as a heterogeneous 67% population (quantity of K160N is about 67%) (Table 1). As previously reported by Wu et al. [37], the K160N substitution results in the formation of additional N-glycosylated forms of HBsAg.

### 3.3. Genetic Characteristics of Samples with Occult Hepatitis B

Genome analysis demonstrated that S-HBsAg from high-copy sample 22, serologically positive for HBeAg, anti-HBeAg, and anti-HBc but negative for HBsAg and anti-HBsAg, had four well-known [36] escape mutations in the a-determinant: T123N, M133T, T140I, and D144E (Table 1). In addition, the antigen contained immunological escape mutations Y100S and V184G [36,39], which are outside the a-determinant but within the MHR (Table 1). According to Mello et al. [40], the Y100C mutation is often observed in OBI. The same sample also included two additional mutations, V118T and V128A. According to Yang et al. [41], the T118M mutant has an impaired antigenicity and makes HBsAg not detectable by ELISA. Koyaweda and co-authors also considered sA128V an escape mutation [39]. The V128T and V128A mutations discovered here may be of a similar significance. Additionally, the T118K mutation is one of the mutations considered critical for immune escape, correlating with HBV reactivation during immunosuppression [25]. Overall, the data indicate that one of the three samples with OBI included eight escape mutations described in the literature. It also contained six additional mutations with an unclear role, although the E164G and G185E substitutions may also belong to escape mutations (Table 1).

Two other samples with OBI traits, having a low content of HBV DNA (samples 53 and 55), were very similar to each other in the spectrum of mutations. Both samples had the mutation profile N40S-G44E-opal69-L89Q-F93Y-L97P-L109Q-S114T-Y134N (Table 1 and Table 5) and differed only in the presence of mutations S113T (sample 55, lymphoma) or D144E (sample 53, AML). Our findings are consistent with the report by Zhang et al. [22] who detected the stop codon opal in the S-HBsAg aa 69 sequence in occult hepatitis B in a patient with genotype C. They are also consistent with the observation that HBsAg is not detected in patients with HBV genotypes C and E with mutations P93Q and T97N/N97T [36], whereas HBV from samples 53 and 55 had F93Y and L97P mutations in S-HBsAg and belonged to genotype D (ayw2). The samples from patients with hematologic cancers are unique not only due to the co-finding of all three mutations affecting the production and detection of HBsAg, but also because of the presence of at least two additional clinically significant escape mutations, L109Q and Y134N [36]. One of those mutations, L109Q, was previously shown to be associated with OBI reactivation in a patient with non-Hodgkin’s lymphoma [42]. Another noteworthy mutation is S114T, since it was found in 18.6% of all tested samples, all of which from patients with leukemias or lymphomas (Table 1, Table 3 and Table 4). Previously, S114T was detected in donors with OBI [43]. Thus, the mutation profile of HBV samples 53 and 55 includes at least six escape mutations.

In total, four samples with mutations at the aa position 69 have been identified, of which three samples included the opal mutation and the fourth sample (sample 52) had a deletion. In contrast to the opal stop codon, the deletion at the aa position 69 did not lead to a decrease in the DNA level. All replacements at the aa position 69 were found in samples with genotype D (ayw2) (Table 1, Table 3, and Table 4).

### 3.4. Mutation Profiles of HBV in Hematological Malignancies

**Figure 1:**
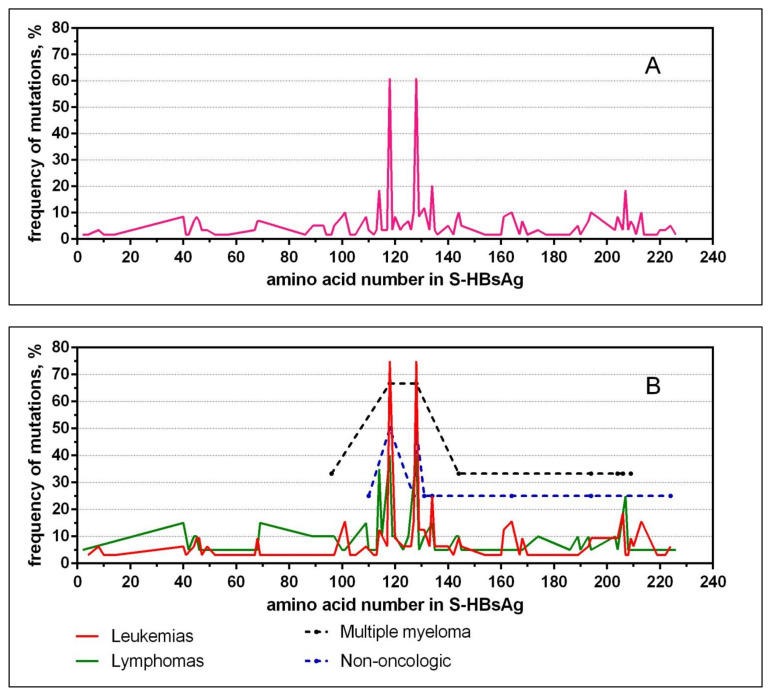
Mutation profile of S-HBsAg in patients with hematologic diseases. (**A**)—for all studied groups, (**B**)—for different hematologic diseases.

The mutation profile of the distribution of aa substitutions within the entire S-HBsAg for all tested samples and for the main experimental groups is presented in Figure 1. The distribution of the total number of mutations in S-HBsAg in the same HBV isolate in different groups of hematological patients is shown in Table 2. The estimated overall frequency of individual aa substitutions in hematological patients is shown in Table 3. The detailed characterization of aa variations and their frequency in different groups of patients are shown in Table 4.

The experiments showed that HBV can simultaneously contain about four different significant escape mutations in the a-determinant, both in patients with leukemia and lymphoma. For example, sample 22 from a patient with CLL simultaneously contained escape mutations T123N-M133T-T140I-D144E, and sample 51 from a patient with B-cell lymphoma contained a set of escape mutations T123N-V125T-T131N-T140I. The occurrence of mutations can be even higher (Table 1 and Table 2). To our knowledge, such a set of mutations has not been described in the literature, although a combination of L109R-C137W-G145R was reported for a patient with advanced non-Hodgkin’s lymphoma, who was probably not vaccinated against HBV and had anti-HBs before immunosuppression as the only possible marker of OBI. This patient developed viremia during immunosuppression (>10^8^ copies/mL) [42]. A similar divergent response, followed by fatal reactivation of hepatitis B, was seen in a patient with B-cell non-Hodgkin’s lymphoma containing escape mutations L109R-Y134S-P142L-D144A, after rituximab therapy [44]. Here, the combinations of the four most well-known mutations in the a-determinant were accompanied by multiple substitutions in the MHR and beyond, including the known cluster mutations [45]. 

Data also showed that substitution at aa 109, which was previously reported upon reactivation of hepatitis B in patients with non-Hodgkin’s lymphoma [42,44], was detected in 5 out of 59 isolates (8.47%) and occurred not only in non-Hodgkin’s lymphoma (two out of 59 isolates, 3.39%), but also in patients with Hodgkin’s lymphoma (one out of 59 isolates, 1.69%) or leukemia (two out of 59 isolates, 3.39%).

Of the 56 tested samples, there were 36 isolates (61.02%) with mutations in S-HBsAg aa 118, including V118T, V118A, and V118M. Moreover, in 100% of cases, these replacements were accompanied by the V128A mutation, indicating a coupled mutation V118T (A, M)/V128A. This mutation was independent of the HBV genotype or subtype, as it was found in samples with genotypes A, D, D2, and D/E that belong to subtypes adw1, adw2, adw3, ayw2, and ayw3, as well as in samples with an undefined subtype.

The second most frequently detected mutation was S114A/P/T. It was seen in 18.6% of cases and only in patients with lymphomas or leukemias. Across lymphoma samples, the frequency of this mutation reached 35%, and it was especially associated with lymphogranulomatosis (six out of seven cases, 85.7% of lymphomas). Incidentally, the adjacent T115N substitution was detected in only two cases (samples 48 and 49), both of which were lymphomas (Table 1 and Table 4). Another adjacent mutation, S113F, was also detected in two cases, of which one was lymphoma (sample 55) and the other was AML (sample 5) (Table 1 and Table 4).

Well-known G145R escape mutation was detected in three samples, two from leukemia patients with CLL and the third from a patient with Hodgkin’s lymphoma. Samples with G145R HBV from patients with CLL (samples 1 and 15) were also similar to each other in the profile of other mutations. Both isolates had mutations in aa 8, 100–101, and 207 and changes in subtyping aa 127 and 134. The samples also contained an escape mutation (M133T or T126N/K). Data also suggest that the G145R mutation may represent a minor population in CLL (quantity is about 25%) (sample 15; Table 1). 

Taken together, the results propose a consensus mutation profile F8L-[Y100C/Q101R]-V118T-T127P/S-V128A-Y134S-G145R-S207R (Table 5). Previously, the reported simultaneous presence of substitutions G145A-F134L was suggested to contribute to the failure of laboratory detection of HBsAg [36].

Another interesting pattern that was also observed was partly associated with the S143T/L substitution. In particular, four samples (4, 18, 29, 33), two of which belong to genotype A (adw1) and two to genotype D (adw1), have a very similar mutation profile, with the 75-100% coincidences (three of four matches are indicated in parentheses): T45S-T46P-T/Y68/I-S114T-V118T-V128A-T131N-Y134F-[S143T]-G159A-F161Y-A168V-[V194A]-[L209V]-L213I (total 15) (Table 5). Of note, samples 4 and 18, both genotypes A of subtype adw1, differ only in the presence of the S143L mutation within the consensus mutation profile, which is absent in ALL (sample 4), but present in CLL (sample 18). This may reflect the degree of the process chronicity. Similarly, samples 29 and 33 of genotype D subtype adw1 differ only in the presence of the V194A mutation, which is present in a patient with Hodgkin’s lymphoma and absent in a patient with AML (Table 5).

It is also likely that the substitutions at aa 143 and aa 145 are accompanied by a replacement at the aa position 207. For example, S143L was accompanied by the S207R/G/N substitution in three out of four cases (75.0%). Similarly, two out of three of isolates with the G145R mutation also had a homogeneous S207R mutation (66.7%). No such pattern was seen for the D144E substitution.

## 4. Discussion

In the Russian population, there is a high incidence of HBV infection among hospitalized patients in hematology departments who were not vaccinated against hepatitis B compared with blood donors [28]. In that study, HBV DNA was detected in 10.1% of the examined patients, while 3.9% of people had occult forms of hepatitis B (negative for HBsAg while positive for HBV DNA). Previously, serologically significant mutations (T115N, G101R, T118V/A128V) in the HBV S-gene were reported in four out of six HBsAg/HBV DNA positive patients [28]. Furthermore, the frequency of aa substitutions identified using the Sanger sequencing was 3.1% of all examined patients or about 30.8% of all infected patients [28].

In another study, when 588 additional hematological Russian patients were examined, it turned out that 4.1% had HBsAg, 7.0 and 15.5% had “isolated” anti-HBc and anti-HBc in combination with anti-HBs, respectively, and 13.9% had anti-HBe in combination with anti-HBs. HBV DNA was detected in 1.4% of HBsAg-negative individuals, which is indicative of occult hepatitis B. Genotyping indicated that 100% of HBV isolates from hematological cancer patients belonged to genotype D. Aa substitutions were detected in 75.0% of infected patients as shown by the Sanger sequencing of the S-gene, which was about 2.0% of the examined patients [21].

Here, occult hepatitis B was found in about 5.08% of all studied patients, which is consistent with previous reports. However, due to the use of NGS, a significantly higher frequency and variability of mutations, as well as their high heterogeneity, were revealed in hematological cancer patients. Indeed, mutations in S-HBsAg were found in 91.5% of all tested samples, while for the group of patients with leukemias and lymphomas, substitutions were detected in 93.4 and 85.0% of cases, respectively. Significantly greater frequencies of mutations, compared with earlier studies, are due to the ability of NGS to detect minor populations of viral variants. Hence, we found that hematological cancer patients are a rather unique group in which HBV mutagenesis proceeds at an extremely high rate, creating multiple mutations of the virus persisting in the patient. Interestingly, in the previous literature, almost each of these mutations was considered an escape mutation. Thus, our research both confirms previous knowledge on and provides further insight into escape mutations and their significance. While nonsynonymous aa substitutions were assessed in the present report, including synonymous substitutions in the analysis would result in a further increase in the mutations frequency. Most of the mutations discovered in this study have clinical significance, as described in the literature for occult hepatitis B, hepatitis B reactivation, and a diagnostic, vaccinal, and immunological escape. This can be a mechanism for the generation of HBsAg molecules with special properties and the emergence of HBV capable of binding to new receptors and infecting different tissues and organs. For example, HBV can infect peripheral blood mononuclear cells (PBMC) [46,47], CD68^+^ cells [48], and CD34^+^ hematopoietic stem cells [49]. The G145R mutant was also found in PBMC [46]. In addition, HBV is known to be an oncogenic virus. It can be assumed that in the presence of predisposing conditions in a patient, such as certain non-malignant blood diseases, HBV infection can increase the risk of oncological transformation. The latter can be particularly relevant if the patient is receiving some hematological-specific therapy while being an OBI carrier.

Results suggest that the mutation process in HBV is initiated at certain positions, which initially has little effect on the patient’s clinical condition but slightly modulates the conformation of the HBsAg molecule. The rearrangement of HBsAg may open additional epitopes that also undergo mutagenesis due to the immune pressure, inducing further mutagenesis in adjacent portions of the molecule. The order of mutations occurrence and their spectrum may depend on the HBV genotype and the patient’s genetic status, as well as the patient’s vaccination status. For the latter, intermediate mutant forms of HBV do not necessarily emerge, and the mutation profile may be different.

The present study also demonstrates that in most samples (61% of isolates), there was a paired mutation at the aa positions 118/128, which allows for the virus to escape the diagnostic control. While this mutation may be nonspecific, it could trigger a further cascade, i.e., being an anchor mutation. In general, mutations that trigger the transition of HBV to OBI can be the first to emerge. Substitutions at the aa positions 114–115, a N40S substitution (8.5% of cases), and, to a lesser extent, substitutions at the aa positions 89–97, as well as the *opal69* mutation are examples of such anchor mutations. According to our data, the S114T mutation was detected in 18.6% of isolates, while in the study by Lin et al. [43], it was frequent in donors with occult hepatitis B. We have shown that mutations N40S, T45S, and T46P are often found in hematological patients with virus genotypes A and D (mutation rates 8.5, 8.5, and 6.8%, respectively), and they co-emerge in different combinations. Others have shown that the N48T substitution (HBV genotype C) causes a decrease in HBsAg detection signals [36]. Substitutions C48G and V96A are localized in class I/II–restricted T-cell epitopes, suggesting their role in HBV evasion from immune T-cells. These substitutions were shown to occur during the reactivation of hepatitis B [25]. A cluster of mutations in the region of 40–45 aa and its relation to the G145R mutation were also reported by Kalinina et al. [45]. Thus, the entire range of substitutions at aa 40–48 may be associated with HBsAg diagnostic defects. Mutations of T-cell escape also include mutations L175S, G185E, and V190A [25]. We were able to detect mutations in 185 and 190 aa, with a frequency of 1.7%. HBV mutants that were reported by Lin et al. [43] were detected in 30.4% of donors with OBI and included S114T, G119R, P120S, T125M, C139Y, T140I, C147W, T148A, A159V/G, E164D, V168A, and R169C. In our study, most of these mutations were also identified, except C139Y, C147W, and T148A.

As a result of the accumulation of certain mutations, the emergence of new glycosylation sites, and escape from the immune system, the virus can gain an advantage in infecting certain tissues, which can determine the type of developing cancer. For example, we found the S114T mutation in 35% of cases with lymphoma. Although Hodgkin’s lymphoma has been linked to the Epstein-Barr virus, HBV may also have a role in the disease.

The results of the current study suggest the significance of the substitutions at the aa positions 129-131 in leukemias. Substitutions Q129N and T131N/M133T in HBsAg interfere with HBV immunogenicity or replication ability [50]. Furthermore, the T131N mutation introduces an additional glycosylation site in the HBsAg molecule [50]. Here, the overall frequency of T131N mutations was 11.9%, and it was more common in patients with leukemias (12.5%). However, this substitution was the only escape mutation in the a-determinant of HBsAg in a patient with MDS, comprising a minor subpopulation of approximately 22%, surrounded by several other mutations such as I110L-V118T-T127P-V128A-T131N-E164G-224G. As MDS is a precancerous condition that can transform into leukemia, this mutation profile may indicate the early stage of the malignant transformation. While in patients with acute leukemia, the number of mutations in HBV increases with the development of the disease, mutations are plentiful in patients with chronic leukemia (Table 1, Table 2, and Table 4 and mutation profile 3 in Table 5).

The assumptions made in this work require further research, accumulation of statistical data, and analysis of HBV mutation profiles in patients over time. The accumulation and classification of experimental data may contribute to a better understanding of the HBV pathogenesis, an identification of the most significant substitutions, and the development of screening diagnostic methods for better patient management. The data indicate the need for extensive monitoring of serological markers and highly sensitive molecular diagnostics of hepatitis B in hospital hematology departments, not only to prevent the spread of mutant forms of the virus, but also in order to choose the correct vaccination strategy and antiviral and immunosuppressive therapy.

Although the S-HBsAg region was the only subject of the genetic analysis in this study, the full-length genome was sequenced for most isolates. Further analysis of the collected data is expected to reveal additional mutations and new patterns in other portions of the HBV genome in hematological cancer patients.

## Figures and Tables

**Table 1:** Serological and genetic characteristics of HBV in 59 isolates from patients with hepatitis B and hematologic diseases ^1^.

Diagnosis	Sample ID	HBsAg	Anti-HBs	HBeAg	Anti-HBe IgG	Anti-HBe IgG + IgM	HBV DNA, Copies/mL	HBV Genotype	HBV Subtype	Amino Acid Substitutions in S-HBsAg	Number of Mutations, n
**Leukemias (n = 32)**											
**Acute leukemia (AL) (n = 4)**	21	+	−	+	−	+	1.6 × 10^8^	D	adw2	V118T, V128A, Q129R, F219S 85%	4
	43	+	−	−	++	+	2.1 × 10^3^	D2	ayw3	V118T, V128A	2
	44	+	−	−	+	+	3.0 × 10^4^	D	ayw2	None	0
	47	+	−	−	+	+	1.6 × 10^2^	D	ayw1	L94S, Y100C, Q101K, L109I, S154Q	5
**Acute lymphoblastic leukemia (ALL) (n = 2)**	54	+	−	+	+	+	3.4 × 10^7^	D	ayw2	M125T, Q129R	2
	56	+	−	−	+	+	2.1 × 10^4^	D2	ayw3	V118A, V128A, V194A	3
**Acute myelogenous leukemia (AML) (n = 12)**	3	+	−	−	n/t ^2^	+	6.8 × 10^2^	D	ayw2/ ayw3	V118T 99%, T127P 35%, V128A 99%, S207R 31%	4
	4	+	n/t	+	n/t	−	7.5 × 10^5^	A	adw1	T45S 98%, T46P 98%, T68I, Y100S, P105R, S114T 99%, V118T, V128A, T126I 77%, T131N 99%, F161Y, A168V, V194A 99%, L209V, L213I 97%	15
	5	+	−	+	+	+	1.6 × 10^7^	D	ayw3	M103I 29%, S113F 27%, S117N 70%, V118A/M 29/71%, T125M 28%, V128A 99%, Q129H 27%, G159A 28%, L213S 62%	10
	8	+	−	+	+	−	7.9 × 10^7^	D	ayw3/adw3	T57I 95%, R122K 66%	2
	12	+	−	+	+	+	9.1 × 10^6^	D	ayw3	I4T 68%, V118T/A, P120T 69%, V128A	5
	24	+	−	+	+	+	n/t	D	ayw3	V117N, V118A, V128A, L213S < 100%	4
	26	+	−	+	+	+	3.8 × 10^7^	D	ayw2	Q101R < 100%, V118T < 100%, V128A, E164G < 100%, S220F < 100%, V224A < 100%	6
	27	+	−	−	+	+	6.8 × 10^8^	D	ayw2	V118T, V128A	2
	28	+	−	−	+	+	4.2 × 10^4^	D	ayw3	N41S, G44E, L49R, P67Q < 100%, V118T, V128A, S204N, S207I/T < 100%, V224A	10
	29	+	−	−	+	+	2.9 × 10^5^	D	adw1	N40S < 100%, T45S, T46P, T68I, S114T, V118T, V128A, T131N, S143T, F161Y, A168V, S204N < 100%, Y206C < 100%, S207G/N < 100%, L209V, S210R, L213I < 100%	18
	32	+	−	+	−	−	4.0 × 10^6^	D	ayw3	V118A, V128A, E164G, F170S	4
	53	−	+	−	+	+	<10^2^	D	ayw2	N40S, G44E, opal69, L89Q, F93Y, L97P, L109Q, S114T, Y134N, D144E	10
**Acute myelogenous monoblastic leukemia (AMML) (n = 2)**	31	+	−	−	+	+	9.5 × 10^2^	D	ayw2	V118T, V128A	2
	42	+	−	+	+	−	1.9 × 10^7^	D	ayw2	V118T, V128A, P142R	3
**Chronic lymphocytic leukemia (CLL) (n = 11)**	1	+	+	−	+	+	1.0 × 10^8^	D/E	ayw2/ayx ^3^	F8L 99%, Q101R 99%, V118T, V128A, M133T 58%, Y134S 37%, G145R, K160N 67%, E164G 38%, S193L, S207R	11
	14	+	−	−	+	+	6.0 × 10^7^	D	ayw2	V118T, V128A, E164A 40%, T189I 21%, S207N/H/R 39%, L222I 21%	8
	15	+	−	−	+	+	1.4 × 10^8^	D	ayw3/aywx	F8L 20%, N52D 29%, Y100C 54%, V118T 100%, T126N/K 61%, T127S 58%, V128A 41%, Y134N 21%, G145R 25%, S204D/G 87%, S207R	13
	17	+	−	−	−	+	1.9 × 10^9^	A	ayw3	None	0
	18	+	−	+	−	−	3.0 × 10^8^	A	adw1	T45S 99%, T46P 94%, T68I 99%, S114T 99%, V118T, P120T 99%, V128A, T131N 99%, S143T 99%, F161Y 99%, A168V, V194A 99%, L209V, L213I 95%	14
	19	+	−	+	−	+	4.3 × 10^8^	D	ayw3/aywx	V118T, V128A, Y134N 89%	3
	20	+	−	−	−	−	8.1 × 10^5^	D	ayw2/aywx	V14A 94%, V47L 92%, V118T 97%, T127P 98%, V128A 97%, Y134F 97%, V177A 85%	7
	22	−	−	+	+	+	10.0 × 10^7^	D	ayw3	G10R, Y100S, V118T, T123N, V128A, M133T 70%, P135L, T140I, D144E, S155F, E164G, S167L, V184G, G185E	14
	23	+	−	+	−	+	1.0 × 10^9^	D	ayw2	V118T, V128A, T140I < 100%	3
	25	+	−	−	+	+	n/t	D	ayw3	L49P < 100%, I86T < 100%, Q101R < 100%, V118A, V128A, T131N 36%, S193L < 100%, I208T, S210R	9
	30	+	−	−	+	+	7.7 × 10^5^	D	x	D144E 72%	1
**Chronic myelogenous leukemia (CML) (n = 1)**	45	+	−	−	−	+	1.8 × 10^7^	x	axx	Q101R, G112R, P120T, R122T, T123I, T\L\P127I, Q129A, Y134N, P135V, F161Y	10
**Lymphomas (n = 20)**											
**Hodgkin’s lymphoma (HL) (n = 12)**	2	+	−	+	±	+	9.4 × 10^7^	D	adw3	E2A 34%, I110N, V118A, V128A, G145R, S174N	6
	9	+	n/t	−	n/t	+	n/t	D	ayw3	S114A 97%	1
	11	+	n/t	n/t	n/t	n/t	n/t	D	adw3	V118A, V128A, S207N, S210R 61%, P211H 59%, F220L 32%	6
	33	+	−	−	+	+	4.5 × 10^8^	D	adw1	T45S, T46P, P67Q, Y68I < 100%, S114T, V118T, V128A, T131N, S143T, F161Y, A168V, L186P, V190F/L < 100%, S193L < 100%, V194A < 100%, P203Q < 100%, S207N, I208T < 100%, L213I/T < 100%	21
	34	+	−	−	+	+	1.3 × 10^1^	D2	ayw3	S114A, T189I < 100%, S193L < 100%, S207R < 100%	4
	35	+	−	−	+	+	3.7 × 10^3^	D	ayw3	S114P < 100%, V118A < 100%, V128A < 100%, S207R < 100%, S143L 31%	5
	36	+	−	+	+	+	4.5 × 10^9^	D2	ayw3	None	0
	37	+	−	+	−	−	2.8 × 10^9^	D2	ayw3	S114A	1
	38	+	−	+	+	+	5.0 × 10^8^	D2	ayw3	None	0
	39	+	n/t	+	n/t	+	n/t	D	ayw2	N40S, opal 69, V118T, P120T 20%, V128A, Q129H, S136F, T189I, P203R	9
	40	+	−	−	+	+	1.1 × 10^5^	D2	ayw3	S114P < 100%	1
	41	+	+	−	+	+	6.3 × 10^7^	D	ayw2	Y100C < 100%, Q101H < 100%, L109I, V118T/M < 100%, G119A/V < 100%, P120T 50%, V128A, D144E 74%, S204N/G < 100%, S207R, P214L	14
**Non-Hodgkin’s lymphoma (NHL) (n = 1)**	10	+	n/t	n/t	n/t	n/t	4.7 × 10^6^	D	adw3	V118T, V128A, D144E, S174N, L222R 66%, I226S 99%	6
**Lymphoma (n = 5)**	46	+	−	−	+	+	2.2 × 10^3^	D	ayw2	None	0
	48	+	−	−	−	−	6.2 × 10^3^	D2	ayw3	T115N	1
	49	+	−	−	−	−	3.0 × 10^3^	D2	ayw3	T115N	1
	52	+	−	+	+	+	3.4 × 10^7^	D	ayw2	N40S, S55F, del 69, L89Q, F93Y, L97P, L109Q, N125T, Y134N	9
	55	−	−	−	−	−	<10^2^	D	ayw2	N40S, G44E, opal 69, L89Q, F93Y, L97P, L109Q, S113T, S114T, Y134N	10
**B-cell lymphoma (n = 1)**	51	+	−	+	+	+	5.1 × 10^2^	D	ayxx	L42R, G44E, T45I, V47A, P56L, G119Q, T123N, V125T, T131N, Y134P, P135T, T140I	12
**T-cell lymphoma (n = 1)**	50	+	−	−	+	+	1.4 × 10^3^	D2	ayw3	V118T, V128A	2
**Multiple myeloma (n = 3)**											
**Multiple myeloma (n = 3)**	7	+	+	n/t	n/t	n/t	8.4 × 10^3^	D	ayw3	V96G 22%, V118A, V128A 93%, S204N 40%, Y206C 31%, L209W 28%	6
	16	+	+	−	+	−	6.3 × 10^8^	D	ayw3	None	0
	59	+	+	−	+	−	3.7 × 10^5^	D2	ayw3	V118T, V128A, D144E, V194A	4
**Non-neoplastic (n = 4)**											
**Myelodysplastic syndrome (MDS) (n = 1)**	6	+	−	+	+	−	2.3 × 10^6^	D	ayw2	I110L 26%, V118T, T127P 89%, V128A, T131N 22%, E164G 30%, V224G 98%	7
**Aplastic anemia (AA) (n = 2)**	13	+	−	+	−	+	1.8 × 10^6^	D2	ayw3	V118A, V128A	2
	58	+	−	−	+	+	2.5 × 10^4^	D	ayw2	V194A	1
**Idiopathic thrombocytopenic purpura (ITP) (n = 1)**	57	+	−	n/t	+	+	1.4 × 10^2^	D	ayw2	Y134N	1

^1^ Allows for heterogeneity for NGS sequenced samples. If there is no % mark, the value is considered 100%; ^2^ n/t—not tested; ^3^ x—cannot be determined.

**Table 2:** Distribution of the total number of S-HBsAg mutations in the same HBV isolate in different groups of patients with hematologic diseases (n = 59).

Disease Group	Frequency of Samples Containing Mutations in S-HBsAg							
	≥1 Mutation		≥5 Mutations		≥10 Mutations		≥15 Mutations	
	Number	%	Number	%	Number	%	Number	%
**Leukemias (n = 32)**	30	93.4	16	50.0	10	31.3	2	6.3
**Lymphomas (n = 20)**	17	85.0	10	50.0	4	20.0	1	5.0
**Multiple myeloma (n = 3)**	2	66.7	1	33.3	−	−	−	−
**Non-cancer diseases (n = 4)**	4	100.0	1	25.0	−	−	−	−

**Table 3:** Overall frequency of individual amino acid substitutions in patients with hematologic diseases (n = 59).

Mutation Frequency, %	Serial Number of an Amino Acid Residue in HBsAg
**1.7**	2, 4, 10, 14, 41, 42, 52, 55, 56, 57, 86, 94, 96, 103, 105, 112, 136, 142, 154, 155, 159, 160, 167, 170, 177, 184, 185, 186, 190, 211, 214, 219, 226
**3.4**	8, 47, 49, 67, 110, 113, 115, 117, 119, 122, 126, 133, 135, 174, 203, 206, 208, 220, 222
**5.1**	89, 93, 97, 123, 140, 145, 189, 210, 224
**6.8**	44, 46, 68, 69, 125, 143, 168, 193, 209
**8.5**	40, 45, 100, 109, 120, 129, 161, 204
**10.2**	101, 127, 144, 164, 194, 213
**11.9**	131
**18.6**	114, 207
**20.3**	134
**61.0**	118, 128

**Table 4:** Detailed characteristics of amino acid variations and their frequency in different groups of patients ^1^.

Aa in S-HBsAg	Variants of			Frequency of aa Substitutions in HBsAg in Clinical Isolates									
	Mutations	Genotypes	Subtypes	Leukemias (n = 32)		Lymphomas (n = 20)		Multiple Myeloma (n = 3)		Non-Cancer Diseases (n = 4)		TOTAL (n = 59)	
				Number	%	Number	%	Number	%	Number	%	Number	%
2	E2A	D	adw3	−	−	1	5.0	−	−	−	−	1	1.7
4	I4T	D	ayw3	1	3.1	−	−	−	−	−	−	1	1.7
8	F8L	D/E, D	ayw2, ayx ^2^, aywx	2	6.3	−	−	−	−	−	−	2	3.4
10	G10R	D	ayw3	1	3.1	−	−	−	−	−	−	1	1.7
14	V14A	D	ayw2	1	3.1	−	−	−	−	−	−	1	1.7
40	N40S	D	adw1, ayw2	2	6.3	3	15.0	−	−	−	−	5	8.5
41	N41S	D	ayw3	1	3.1	−	−	−	−	−	−	1	1.7
42	L42R	D	ayxx	−	−	1	5.0	−	−	−	−	1	1.7
44	G44E	D	ayw2, ayw3, ayx	2	6.3	2	10.0	−	−	−	−	4	6.8
45	T45S, T45I	A, D	adw1, ayx	3	9.4	2	10.0	−	−	−	−	5	8.5
46	T46P	A, D	adw1	3	9.4	1	5.0	−	−	−	−	4	6.8
47	V47A	D	ayw2, ayx	1	3.1	1	5.0	−	−	−	−	2	3.4
49	L49R	D	ayw3	2	6.3	−	−	−	−	−	−	2	3.4
52	N52D	D	aywx	1	3.1	−	−	−	−	−	−	1	1.7
55	S55F	D	ayw2	−	−	1	5.0	−	−	−	−	1	1.7
56	P56L	D	ayxx	−	−	1	5.0	−	−	−	−	1	1.7
57	T57I	D	adw3	1	3.1	−	−	−	−	−	−	1	1.7
67	P67Q	D	adw1, ayw3	1	3.1	1	5.0	−	−	−	−	2	3.4
68	T68I, Y68I	A, D	adw1	3	9.4	1	5.0	−	−	−	−	4	6.8
69	Deletion, *opal*	D	ayw2	1	3.1	3	15.0	−	−	−	−	4	6.8
86	I86T	D	ayw3	1	3.1	−	−	−	−	−	−	1	1.7
89	L89Q	D	ayw2	1	3.1	2	10.0	−	−	−	−	3	5.1
**93**	F93Y	D	ayw2	1	3.1	2	10.0	−	−	−	−	3	5.1
**94**	L94S	D	ayw1	1	3.1	−	−	−	−	−	−	1	1.7
**96**	V96G	D	ayw3	−	−	−	−	1	33.3	−	−	1	1.7
**97**	L97P	D	ayw2	1	3.1	2	10.0	−	−	−	−	3	5.1
**100**	Y100S, Y100C	A, D	adw1, ayw1, ayw2, ayw3, aywx	4	12.5	1	5.0	−	−	−	−	5	8.5
101	Q101H, Q101R, Q101K	D/E, D, X	ayw1, ayw2, ayw3, x	5	15.6	1	5.0	−	−	−	−	6	10.2
**103**	M103I	D	ayw3	1	3.1	−	−	−	−	−	−	1	1.7
105	P105R	A	adw1	1	3.1	−	−	−	−	−	−	1	1.7
**109**	L109I, L109Q	D	ayw1, ayw2	2	6.3	3	15.0	−	−	−	−	5	8.5
**110**	I110N, I110L	D	adw3, ayw2	−	−	1	5.0	−	−	1	25.0	2	3.4
112	G112R	X	x	1	3.1	−	−	−	−	−	−	1	1.7
113	S113F, S113T	D	ayw2, ayw3	1	3.1	1	5.0	−	−	−	−	2	3.4
**114**	S114A, S114P, S114T	A, D, D2	adw1, ayw2, ayw3	4	12.5	7	35.0	−	−	−	−	11	18.6
115	T115N	D2	ayw3	−	−	2	10.0	−	−	−	−	2	3.4
117	S117N, S117V	D	ayw3	2	6.3	−	−	−	−	−	−	2	3.4
**118**	V118A, V118T, V118M	A, D, D2, D/E	adw1, adw2, adw3, ayw2, ayw3, ayx	24	75.0	8	40.0	2	66.7	2	50.0	36	61.0
**119**	G119A, G119V, G119Q	D	ayw2, ayx	−	−	2	10.0	−	−	−	−	2	3.4
**120**	P120T	A, D, X	adw1, ayw2, ayw3, ax	3	9.4	2	10.0	−	−	−	−	5	8.5
**122**	R122K, R122T	A, D, X	adw3, ayw3, x	2		−	−	−	−	−	−	2	3.4
**123**	T123N/I	D, X	ayw3, ayx, x	2	6.3	1	5.0	−	−	−	−	3	5.1
**125**	T125M, V125T, N125T, M125T	D	ayw2, ayw3, ayx	2	6.3	2	10.0	−	−	−	−	4	6.8
**126**	T126I, T126N/K	A, D	adw1, aywx	2	6.3	−	−	−	−	−	−	2	3.4
**127**	T127S, T127P, T\L\P127I	D, X	ayw2, ayw3, aywx, x	5	15.6	−	−	−	−	1	25.0	6	10.2
**128**	V128A	A, D, D2, D/E	adw1, adw2, adw3, ayw2, ayw3, ayx	24	75.0	8	40.0	2	66.7	2	50.0	36	61.0
**129**	Q129H, Q129R, Q129A	D, X	adw2, ayw2, ayw3, x	4	12.5	1	5.0	−	−	−	−	5	8.5
**131**	T131N	A, D	adw1, ayw2, ayw3, ayx	4	12.5	2	10.0	−	−	1	25.0	7	11.9
**133**	M133T	D, D/E	ayw2, ayw3, ayx	2	6.3	−	−	−	−	−	−	2	3.4
**134**	Y134S, Y134F, Y134N, Y134P	A, D, D/E, X	adw1, ayw2, ayw3, aywx ayx x	8	25.0	3	15.0	−	−	1	25.0	12	20.3
135	P135L, P135V, P135T	D, X	ayw3, ayx, x	2	6.3	1	5.0	−	−	−	−	2	3.4
136	S136F	D	ayw2	−	−	1	5.0	−	−	−	−	1	1.7
**140**	T140I	D	ayw2, ayw3, ayx	2	6.3	1	5.0	−	−	−	−	3	5.1
**142**	P142R	D	ayw2	1	3.1	−	−	−	−	−	−	1	1.7
**143**	S143T, S143L	A, D	adw1, ayw3	2	6.3	2	10.0	−	−	−	−	4	6.8
**144**	D144E	D, D2	adw3, ayw2, ayw3, x	3	9.4	2	10.0	1	33.3	−	−	6	10.2
**145**	G145R	D, D/E	adw3, ayw2, aywx, ayx	2	6.3	1	5.0	−	−	−	−	3	5.1
154	S154Q	D	ayw1	1	3.1	−	−	−	−	−	−	1	1.7
155	S155F	D	ayw3	1	3.1	−	−	−	−	−	−	1	1.7
**159**	G159A	D	ayw1, ayw3	1	3.1	−	−	−	−	−	−	1	1.7
**160**	K160N	D/E	ayw2, ayx	1	3.1	−	−	−	−	−	−	1	1.7
**161**	F161Y	A, D, X	adw1, x	4	12.5	1	5.0	−	−	−	−	5	8.5
**164**	E164G, E164A	D, D/E	ayw2, ayw3, ayx	5	15.6	−	−	−	−	1	25.0	6	10.2
167	S167L	D	ayw3	1	3.1	−	−	−	−	−	−	1	1.7
**168**	A168V	A, D	adw1	3	9.4	1	5.0	−	−	−	−	4	6.8
170	F170S	D	ayw3	1	3.1	−	−	−	−	−	−	1	1.7
174	S174N	D	adw3	−	−	2	10.0	−	−	−	−	2	3.4
177	V177A	D	ayw2, ayw3	1	3.1	−	−	−	−	−	−	1	1.7
**184**	V184G	D	ayw3	1	3.1	−	−	−	−	−	−	1	1.7
**185**	G185E	D	ayw3	1	3.1	−	−	−	−	−	−	1	1.7
186	L186P	D	adw1	−	−	1	5.0	−	−	−	−	1	1.7
**189**	T189I	D, D2	ayw2, ayw3	1	3.1	2	10.0	−	−	−	−	3	5.1
**190**	V190F, V190L	D	adw1	−	−	1	5.0	−	−	−	−	1	1.7
193	S193L	D, D2, D/E	adw1, ayw2, ayw3, ayx	2	6.3	2	10.0	−	−	−	−	4	6.8
194	V194A	A, D, D2	adw1, ayw2, ayw3	3	9.4	1	5.0	1	33.3	1	25.0	6	10.2
203	P203Q, P203R	D	adw1, ayw2	−	−	2	10.0	−	−	−	−	2	3.4
204	S204N, S204D, S204G	D	adw1, ayw2, ayw3, aywx	3	9.4	1	5.0	1	33.3	−	−	5	8.5
**206**	Y206C	D	adw1, ayw3	1	3.1	−	−	1	33.3	−	−	2	3.4
**207**	S207R, S207N, S207H, S207I, S207T, S207G	D, D2, D/E	adw1, adw3, ayw2, ayw3, aywx, ayx	6	18.8	5	25.0	−	−	−	−	11	18.6
208	I208T	D	adw1, ayw3	1	3.1	1	5.0	−	−	−	−	2	3.4
209	L209V, L209W	A, D	adw1, ayw3	3	9.4	−	−	1	33.3	−	−	4	6.8
210	S210R	D	adw1, adw3, ayw3	2	6.3	1	5.0	−	−	−	−	3	5.1
211	P211H	D	adw3	−	−	1	5.0	−	−	−	−	1	1.7
213	L213I, L213S, L213T	A, D	adw1, ayw3	5	15.6	1	5.0	−	−	−	−	6	10.2
214	P214L	D	ayw2	−	−	1	5.0	−	−	−	−	1	1.7
219	F219S	D	adw2	1	3.1	−	−	−	−	−	−	1	1.7
**220**	F220L, S220F	D	adw3, ayw2	1	3.1	1	5.0	−	−	−	−	2	3.4
222	L222R, L222I	D	adw3, ayw2, ayw3	1	3.1	1	5.0	−	−	−	−	2	3.4
224	V224G, V224A	D	ayw2, ayw3	2	6.3	−	−	−	−	1	25.0	3	5.1
226	I226S	D	adw3	−	−	1	5.0	−	−	−	−	1	1.7

^1^ The major hydrophilic region (MHR) (99–169 aa) is highlighted in gray, and the a-determinant (aa 124–147) within the MHR is highlighted in dark gray [36]. Amino acid (aa) positions in S-HBsAg with established clinical significance according to literature data are shown in bold. ^2^ x—cannot be determined.

**Table 5:** Mutation profiles (linked mutations) of HBV in hematological cancer patients.

Profile	Sample	Genotype	Subtype	Disease	Common Similar or Closely Related Mutations in S-HBsAg
**1**	1	D/E	ayw2/ayx ^1^	Leukemia (CLL)	F8L-[Y100C]-V118T-T127P-V128A-Y134S-G145R-S207R
	15	D	aywx	Leukemia (CLL)	F8L-[Q101R]-V118T-T127S-V128A-Y134N-G145R-S207R
**2**	53	D	ayw2	Leukemia (AML)	N40S-G44E-*opal*69-L89Q-F93Y-L97P-L109Q-S114T-Y134N
	55	D	ayw2	Lymphoma	N40S-G44E-*opal*69-L89Q-F93Y-L97P-L109Q-S114T-Y134N
**3**	4	A	adw1	Leukemia (AML)	T45S-T46P-T68I-S114T-V118T-V128A-T131N-Y134F-F161Y-A168V-V194A-L209V-L213I
	18	A	adw1	Leukemia (CLL)	T45S-T46P-T68I-S114T-V118T-V128A-T131N-Y134F-S143T-F161Y-A168V-V194A-L209V-L213I
	29	D	adw1	Leukemia (AML)	T45S-T46P-T68I-S114T-V118T-V128A-T131N-Y134F-S143T-F161Y-A168V-L209V-L213I
	33	D	adw1	Hodgkin’s lymphoma (HL)	T45S-T46P-Y68I-S114T-V118T-V128A-T131N-Y134F-S143T-F161Y-A168V-V194A-L209V-L213I/T

^1^ x—cannot be determined.
